# Immune Cell Therapies to Improve Regeneration and Revascularization of Non-Healing Wounds

**DOI:** 10.3390/ijms21155235

**Published:** 2020-07-23

**Authors:** Elena Groppa, Andrea Colliva, Roman Vuerich, Tea Kocijan, Serena Zacchigna

**Affiliations:** 1 Cardiovascular Biology Laboratory, International Centre for Genetic Engineering and Biotechnology (ICGEB), 34149 Trieste, Italy; groppa@icgeb.org (E.G.); colliva@icgeb.org (A.C.); vuerich@icgeb.org (R.V.); kocijan@icgeb.org (T.K.); 2 Department of Medical, Surgical and Health Sciences, University of Trieste, 34127 Trieste, Italy; 3 Department of Life Sciences, University of Trieste, 34127 Trieste, Italy

**Keywords:** wound healing, cell therapy, animal models, clinical trials

## Abstract

With the increased prevalence of chronic diseases, non-healing wounds place a significant burden on the health system and the quality of life of affected patients. Non-healing wounds are full-thickness skin lesions that persist for months or years. While several factors contribute to their pathogenesis, all non-healing wounds consistently demonstrate inadequate vascularization, resulting in the poor supply of oxygen, nutrients, and growth factors at the level of the lesion. Most existing therapies rely on the use of dermal substitutes, which help the re-epithelialization of the lesion by mimicking a pro-regenerative extracellular matrix. However, in most patients, this approach is not efficient, as non-healing wounds principally affect individuals afflicted with vascular disorders, such as peripheral artery disease and/or diabetes. Over the last 25 years, innovative therapies have been proposed with the aim of fostering the regenerative potential of multiple immune cell types. This can be achieved by promoting cell mobilization into the circulation, their recruitment to the wound site, modulation of their local activity, or their direct injection into the wound. In this review, we summarize preclinical and clinical studies that have explored the potential of various populations of immune cells to promote skin regeneration in non-healing wounds and critically discuss the current limitations that prevent the adoption of these therapies in the clinics.

## 1. The Clinical Problem

The skin is the largest organ in mammals, which shields the organism from thermal, mechanical, and chemical damage; toxins; and microorganisms, serving as a protective barrier at the interface between the human body and the surrounding environment. Loss of skin integrity often results in physiologic imbalance and disability, or even death, with significant impact on the healthcare system [1,2]. Healing of any wound requires a coordinated response of multiple cell types, either residing in the various skin layers (epidermis and dermis) or recruited from the circulation [3]. This can be schematically divided in an early inflammatory phase, followed by a proliferative and regenerative phase, associated with massive extracellular matrix (ECM) remodeling [3,4].

This process is often compromised in common diseases that are highly prevalent in the aged population and include diabetes, obesity, and vascular disorders. As such, the incidence of chronic non-healing wounds is progressively increasing. Recent studies report a general prevalence of non-healing wounds of approximately 1–2%, similar to the one of heart failure [5,6,7].

Clinically, a chronic wound is defined as a full-thickness skin defect that fails to heal after 3 months of standard care [8]. Chronic wounds are generally classified into vascular ulcers (caused by either venous or arterial diseases), diabetic ulcers, and pressure ulcers. Additional, less common, etiologies include infections [9], immune diseases [10,11], traumas [12,13], large-area burns [14,15], and post-surgery complications [4,16,17,18]. Despite recent progresses in revascularization procedures and in the use of dermal and epidermal substitutes to improve healing, these ulcers often persist for more than one year and tend to recur in up to 70% of patients, significantly compromising their quality of life [19,20]. In 2020, the rate of both minor and major limb amputations is still very high, with reported incidences of 14–24% in patients with foot ulcers over the last decades [8].

Despite a heterogeneous etiology, most chronic wounds are characterized by persistent inflammation [20]. Excessive levels of inflammatory cytokines, proteolytic enzymes, and reactive oxygen species (ROS) damage both cellular and extracellular components, creating a proinflammatory feedback loop [20,21] Moreover, proteases degrade both growth factors and their receptors, resulting in cell cycle arrest and inhibition of proliferation and regeneration [22,23,24].

A common feature of all chronic wounds is insufficient vascularization, which represents a major impediment to healing and regeneration [25,26]. Appropriate supply of nutrients and growth factors, as well as maintenance of oxygen homeostasis, are essential to support cell proliferation, migration, and recruitment of immune cell populations [3,27], which play a fundamental role in both phases of the healing process.

**Figure 1:**
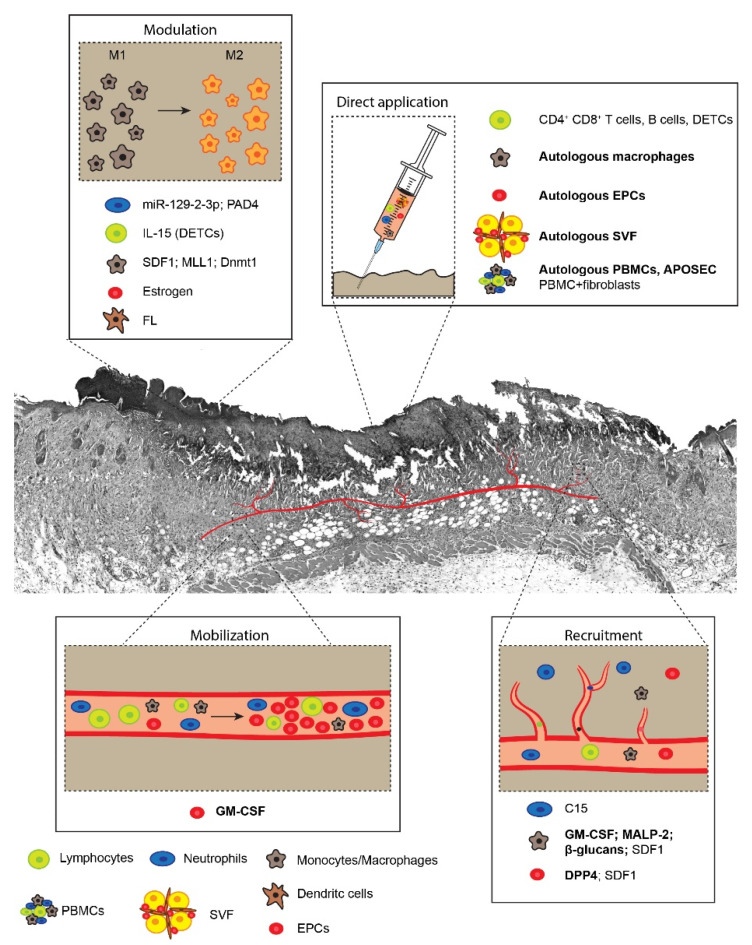
The figure shows examples of cellular therapies proposed and exploited for the treatment of non-healing wounds, grouped according to four main approaches: cell mobilization into the circulation, cell recruitment to the wound site, modulation of cell function/activity, and direct application of cells to the wound site. Studies that reached the clinical stage are in bold. APOSEC, apoptotic secretome; C15, chemerin 15; DETC, dendritic epidermal T cells; Dnmt1, DNA (cytosine-5)-methyltransferase 1; DPP4, dipeptidyl peptidase-4; EPCs, endothelial progenitor cells; FL, FMS-like tyrosine kinase-3 ligand; GM-CSF, granulocyte-macrophage colony stimulating factor; IL-15, interleukin-15; MALP2, macrophage-activating lipopeptide-2; MLL1, mixed-lineage leukemia 1; PAD4, peptidyl-arginine deiminase 4; PBMCs, peripheral blood mononucleated cells; SDF1, stromal cell-derived factor-1; SVF, stromal vascular fraction.

The following chapters describe the existing models, commonly used to evaluate the efficacy of innovative therapies for skin regeneration. In particular, we consider various immune cell types, belonging to both innate and adaptive immunity, that contribute to wound healing and have been considered for their therapeutic potential in either preclinical or clinical studies. These therapeutic strategies rely on (i) immune cell mobilization into the circulation, (ii) cell recruitment and homing at the level of the skin, (iii) modulation of their pro-regenerative activity, and (iv) direct cell implantation to the wound site. Although we could not include all publications available in this expanding field, representative studies supporting each of these approaches have been cited. The most relevant approaches are schematically shown in Figure 1 and further details are provided in Table 1.

## 2. Animal Models of Wound Healing

Both small and large animal models have been developed to assess the regenerative potential of immune cells in wound healing [61]. Obvious differences in the anatomy and physiology of the skin in different species justify the use of multiple models, as no single one can reliably reproduce the clinical situation [61,62], and, more importantly, the comorbidities that are often responsible for chronic wounds [63,64].

Because chronic wounds usually occur in people affected by diabetes, ischemia, and mechanical pressure [8,65], these conditions are generally induced in animals, prior to the generation of an acute wound [66,67,68,69].

Mice are the most common species used for this purpose, as their housing is relatively cheap and affordable by most laboratories, they can be genetically manipulated for mechanistic investigation, and state-of-the-art protocols to induce co-morbidities (i.e., diabetes and ischemia) are available [70]. Yet, the mechanism of wound closure is very different in mice and humans. While murine wounds essentially close by contraction, in humans, wound healing relies on the formation of a new tissue, composed by tightly packed fibroblasts, keratinocytes, and endothelial and inflammatory cells (granulation tissue), which is progressively reabsorbed and replaced by re-epithelialization [71]. Preferential healing by contraction in rodents is mainly due to the presence of a thick layer of subcutaneous striated muscle tissue (named *panniculus carnosus*), absent in humans, and by the presence of active myofibroblasts. Several strategies have been developed to limit wound contraction in mice (i.e., the application of either a silicone splint around the wound or a dorsal skinfold chamber), allowing for the formation of granulation tissue and re-epithelialization, similar to what happens in human wound healing [71,72,73]. Combined with intravital microscopy, the dorsal skin fold chamber allows high-resolution real-time imaging of re-vascularization during skin regeneration [74]. While not yet used at a large extent, the possibility to monitor the formation of new blood vessels appears critical to develop new therapies improving wound healing and predict their efficacy in the clinics, as the whole skin regeneration requires an appropriate supply of nutrients and oxygen [75].

The evaluation of in vivo efficacy and safety of human cell therapies requires the use of immunocompromised mice, i.e., NSG (NOD scid gamma) mice, which lack B and T lymphocytes, as well as natural killer (NK) cells [44,76,77,78]. These mice are extensively used in the preclinical assessment of human cell-based therapies, as they allow the engraftment of human cells, but obviously do not recapitulate the complex immune response required for wound healing. 

In conclusion, mice are very useful for the dissection of genetic, cellular, and molecular mechanisms of skin regeneration, but they cannot be considered the gold standard for therapeutic applications. Pigs share significant anatomical and physiological similarities to humans, in terms of epidermal and dermal thickness and structure, dense dermal collagen and elastin fibers, epidermal turnover time (approximately 30 days), pattern and structure of hair follicles, and biological response to growth factors [61,79,80]. Moreover, the mechanism of wound healing is very similar in pigs and humans, relying on the formation of granulation tissue, followed by re-epithelialization with minimal skin contraction [64]. Finally, the colonization of the porcine skin by immune cells is quite comparable to the human and different from the murine one, as also discussed later in more detail [79,80,81,82,83]. Because of these numerous similarities, pigs are considered the gold standard in the transition from preclinical to human studies. Yet, their use is importantly limited by high costs, absence of transgenic models for cell tracing, and a need for both sophisticated instrumentation and skilled personnel [64,79]. 

## 3. Neutrophils

Neutrophils are part of the innate immune system; they do not physiologically reside in the skin, but they are rapidly recruited to the wound site after injury [21,84]. They produce antimicrobial substances and proteases, most of which are contained in their cytoplasmic granules. They also generate cytokines and growth factors to recruit other components of the inflammatory system and support angiogenesis, keratinocyte, and fibroblast proliferation. Upon activation, neutrophils release proteins and decondensed chromatin that together form extracellular fibers able to trap bacteria. These neutrophil extracellular traps (NETs) degrade virulence factors and kill bacterial cells [85]. While their beneficial role is exerted during the early inflammatory phase of wound healing, their prolonged persistence caused by extensive damage and/or ongoing microbial contamination can inhibit the late reparative phase due to continuous release of proteases that degrade the ECM [86]. Thus, inhibition of their persistent activity has been proposed as a therapeutic strategy in preclinical experimentation, although it has not been translated to the clinics yet.

In an attempt to identify the molecular determinants of persistent neutrophil activation in diabetic conditions, Umehara and colleagues investigated specific miRNAs that are differentially expressed by neutrophils in diabetic and healthy mice and identified miR-129-2-3p as one of the most downregulated [28]. Consistent with the capacity of this miRNA to inhibit the expression of proinflammatory genes (i.e., Caspase-6 and C-C chemokine receptor type 2, Ccr2), its topical application to a wound of diabetic mice resulted in improved healing, although the cell type responsible for this in vivo effect has not been clearly identified. 

Furthermore, neutrophils isolated from fresh whole blood obtained from diabetic individuals produce an increased amount of NETs [29]. High glucose correlates with elevated calcium levels in neutrophils and enhanced activation of calcium-dependent enzymes that are essential for chromatin decondensation (i.e., peptidylarginine deiminase 4, PAD4). Consistently, genetic inactivation of PAD4 or systemic NETosis inhibition by DNaseI improved wound healing and re-epithelialization in diabetic mice. 

As any inflammatory cell, neutrophils are recruited to the wound by chemoattractant molecules that often bind receptors expressed by multiple cell types, thus allowing a coordinated multicellular response. One example is ChemR23, a receptor abundantly expressed by neutrophils but also by platelets and macrophages. This receptor binds the anti-inflammatory and pro-resolving peptide Chemerin15 (C15), which is derived from the cleavage of the chemoattractant protein chemerin, mostly known for its role in adipocyte differentiation and lipolysis [87]. C15 exerts reduced chemoattractive and enhanced anti-inflammatory activities compared to full-length chemerin [88]. Accordingly, its topical delivery as a gel on cutaneous burn wounds reduced the extension of granulation tissue, accelerating closure and re-epithelialization [30], as measured by fluorescence spinning disk intravital imaging. These data are consistent with previous evidence showing that C15/ChemR23 signaling suppresses integrin clustering in neutrophils, thereby reducing their trans-endothelial migration [89]. 

## 4. Monocytes/Macrophages

As in any response to tissue injury, circulating monocytes are massively recruited to the site of the skin wound, where they differentiate into macrophages able to mediate both destructive and reparative functions [90,91]. Various monocyte subtypes can be recognized based on the expression level of multiple cell surface markers. During the early phase of wound healing, CD11b^+^/Ly6C^hi^ cells, commonly named M1 macrophages, produce abundant levels of proinflammatory molecules, including IL-1β, TNF-α, IL-6, and iNOS (inducible NO synthase) [92,93]. During the second phase, proinflammatory M1 macrophages switch toward an anti-inflammatory and pro-regenerative M2 phenotype (CD11b^+^/Ly6C^lo^), characterized by the secretion of Transforming Growth Factor (TGF)-β1, Vascular Endothelial Growth Factor (VEGF)-A, Arginase-1, and IL-10 [93,94]. As any classification, the distinction between M1 and M2 macrophages appears to be over simplistic. Macrophages recruited during the early phase sometimes express M2 markers and both macrophage types derive from the same pool of circulating monocytes [95,96]. Thus, it is very difficult, if not unrealistic, to strictly control macrophage polarization for therapeutic purposes, as highlighted in recent reviews [90,92,97].

In pathological states, like diabetes and obesity, monocyte recruitment, their differentiation into macrophages, and macrophage polarization are often impaired [93,98]. Hypertrophic adipocytes produce a plethora of adipokines that attract M1 macrophages and create a feedforward loop, resulting in a chronic low-grade inflammatory state [99,100] and lack of transition to an effective reparative phase [93,98].

Macrophage-based cell therapies that have reached clinical experimentation rely on their recruitment, activity modulation, or direct injection into the wound [97]. The most exploited strategy has been the local injection of human granulocyte-macrophage colony stimulating factor (hGM-CSF), which promoted healing in both refractory chronic skin ulcers and second-degree burns [32,33,34]. Approaches so far considered for their capacity to activate macrophages are based on the use of compounds that are naturally produced by microorganisms and can be purified as clinical-grade molecules. Among these are β-glucans, natural components of the cell walls of bacteria and fungi, which constitute the principal components of several Asiatic medicinal products, and the mycoplasma-derived macrophage-activating lipopeptide-2 (MALP-2) [35,36]. The local application of these immunomodulators has been reported as safe and potentially useful to improve healing of human wounds, although further studies will be needed to definitely prove efficacy [35,36]. Finally, the local injection of autologous macrophages has been attempted to treat both deep sternal wound infections following open heart surgery and refractory human ulcers in diabetic feet [37,38]. While these studies confirm the feasibility of the approach, no conclusions can be drawn in terms of safety and efficacy.

Because the initial expectations raised by these cellular therapies have not been met by the results of clinical studies, more preclinical work is needed to better define the appropriate timing of macrophage administration [101] and the mechanisms by which they could be of real benefit in skin regeneration. Over the last six years, additional animal studies have been undertaken to identify novel molecules able to boost both macrophage recruitment and polarization. Perhaps the most promising one is CXCL12 (SDF1), which recruits macrophages through its binding to CXCR4. The local application of lactic acid bacteria transformed with a CXCL12-expressing plasmid accelerated wound closure in healthy mice, in mouse models of hyperglycemia and peripheral ischemia, as well as in an ex vivo model of human skin [31]. In addition, exposure to CXCL12 favored M2 polarization and secretion of TGF-β. Similar results on macrophage polarization were obtained by using exosomes derived from a macrophage cell line (Raw264.7) [102]. Macrophage polarization is known to depend on multiple epigenetic changes, which are often impaired in diabetes [39,40]. At least two methyltransferases, MLL1 and Dnmt1, appear upregulated in macrophages of diabetic patients, in which they impose an activation mark on the promoter of proinflammatory genes (i.e., IL-1β, NOS2, and TNF-α) and inhibit the expression of factors inducing macrophage differentiation, respectively. The genetic inactivation of these methyltransferases has been shown to improve wound healing in animal models. However, the translation of this approach to humans is still unproven.

In conclusion, several open questions remain, which at present do not support the clinical use of these cells. First, the correlation between the mouse and human macrophage phenotype has been poorly proven. Second, the appropriate timing for macrophage therapy application has not been defined either in preclinical or clinical settings. Finally, as also highlighted for other cell populations, the design of clinical trials has been so far of poor quality, in terms of both patient recruitment and clinical endpoints.

## 5. Dendritic Cells

Dendritic cells (DCs) are antigen presenting cells that prime T cell responses. In the epidermis, DCs are called Langerhans cells (LCs) and derive their name from the surface receptor CD207 (Langerin). They exhibit a unique behavior, characterized by rhythmic extension and retraction of their dendrites in intercellular spaces between keratinocytes [3,103]. In the dermis, there are two subtypes of resident DCs, also named type 1 and type 2 myeloid/conventional DCs, which trigger Th1 and Th2 responses, respectively [104,105]. Epidermal and dermal DCs serve as first-line defenders, presenting new antigens to T cells within the dermis and migrating into draining lymph nodes where they continue to activate T cell-mediated adaptive responses [3,106].

In addition, plasmacytoid dendritic cells (pDCs) do not physiologically reside in the skin but are recruited together with neutrophils in case of injury [107]. They are particularly active during the early phase, when they produce IFN-α/β in response to nucleic acids released by damaged cells and sensed by the intracellular toll-like receptor 7 and 9 (TLR7 and TLR9). 

Preclinical evidence of their positive role stems from multiple animal models of DC depletion generated using either transgenic animals or neutralizing antibodies. DC depletion invariably resulted in a suppressed early inflammatory response, reduced re-epithelialization, and decreased revascularization of healing wounds [41,107]. Conversely, systemic treatment with the DC-specific growth factor recombinant FMS-like tyrosine kinase-3 ligand (FL) significantly accelerated wound closure when delivered during the early but not the late phase of wound healing. Despite these promising insights into the role of DCs in wound healing, their therapeutic relevance in humans is still completely unexplored.

## 6. Lymphocytes

The adaptive immune response is orchestrated by lymphocytes, a heterogeneous population that include B cells, responsible for the humoral response, and T cells, which mainly exert cytotoxic activity. While their major function is to combat pathogen invasion, emerging evidence points toward their role in regenerative processes, including wound healing [108]. The healthy skin is colonized by different T cell subsets, identifiable by the expression of specific T cell receptor (TCR) isoforms. Two major populations, namely γδ-TCR and αβ-TCR lymphocytes, physiologically reside in the skin of both humans and rodents [109].

The most abundant T cell types in the murine healthy skin are two specialized classes of γδ-TCR lymphocytes, namely Vγ5Vδ1 dendritic epidermal T cells (DETCs) and Vγ4 dermal lymphocytes [110]. The crucial role of γδ-TCR lymphocytes in wound healing has been first described using TCRδ^−/−^mice, which lack epidermal lymphocytes and show a poor skin regenerative response after injury, resulting in reduced keratinocyte proliferation [111,112]. 

DETCs stably reside in the epidermis and have a typical dendritic cell shape, which facilitates their interaction with keratinocytes [113]. Upon activation by infection or injury, they change into round-shaped active T cells and promote keratinocyte proliferation through the secretion of keratinocyte growth factor (KGF-1) and insulin-like growth factor-1 (IGF-1) [114]. Vγ4 dermal lymphocytes normally reside in the dermis and migrate to the epidermis in response to proinflammatory molecules, such as chemokine (C-C motif) ligand 20 (CCL20), which binds chemokine receptor 6 (CCR6) expressed on their membrane [115,116]. These cells participate in the first phase of wound healing through the secretion of tumor necrosis factor α (TNF-α) and IL-17 [117].

Therapeutic approaches exploit a positive feedback loop existing between DETCs and keratinocytes. Injured keratinocytes secrete IL-15, which stimulates IGF-1 secretion by DETCs [118]. In turn, IGF-1 promotes keratinocyte proliferation and further secretion of IL-15. The importance of this paracrine loop in skin regeneration has been demonstrated in diabetic mice, which have reduced levels of IL-15 and a low number of DETC in their epidermis, with consequent decreased levels of KGF-1 and IGF-1 and impaired wound healing. Engraftment of an extra amount of DETCs in the same mice rescued the KGF-1 and IGF-1 levels and improved wound regeneration [43,118]. A similar therapeutic effect was exerted by the administration of IL-15, which restored IGF-1 production by DETC both in vivo and in vitro [42].

In addition to DETC, Vγ4 T cells are also reduced in diabetic mice, consistent with decreased levels of both IL-17 and CCL20 [119]. Evidence supporting the role of Vγ4 T cells in skin regeneration comes from IL-17^−/−^mice, which are characterized by defective wound healing [120], as well as from the therapeutic activity of IL-17 in improving wound healing in diabetic mice [119]. More recently, conflicting data have been reported, showing that IL-17 inhibits IGF-1 secretion in the wound bed and that Vγ4 T cell depletion accelerates wound healing, which would indicate a negative effect of these cells in the process [116].

Additional concerns on the therapeutic potential of γδ-TCR lymphocytes for regenerative medicine stems from the fact that no human counterpart for either DETC or Vγ4 T cells has been clearly identified and that γδ-TCR cells represent less than 10% of total T cells in the human skin [121]. One single study reported the capacity of a specific subset of human γδ-TCR cells to produce IGF-1 in vitro when isolated from an acute but not from a chronic wound [122]. 

The αβ-TCR lymphocytes (CD4^+^ helper and CD8^+^ cytotoxic) represent the vast majority of skin-resident T cells in humans, and a consistent part of them in mice [110,122]. While in humans, they reside in the epidermis, and in mice, they are localized mainly in the dermis, proximal to hair follicles [123,124]. In addition to the resident population, circulating CD4^+^ and CD8^+^ lymphocytes are recruited to the wound bed, peaking at day 1 and 7, respectively. They exert antimicrobial function during the early inflammatory phase and recruit additional immune cells (neutrophils and macrophages) through the secretion of a variety of cytokines, including IL-1β, IL-6, TNF-α, CXCL2, and CCL2 [108,125]. Several studies highlighted the detrimental role of CD4^+^ and CD8^+^ lymphocytes in tissue regeneration and revascularization. For instance, CD4^+^ cell ablation promotes heart regeneration [126] and both CD4^+^ [127] and CD8^+^ [128] blockade improves angiogenesis in diabetic mice. Yet, their functional role in skin regeneration is controversial, as indicated by numerous studies reporting conflicting results, and a clear discrimination of the specific role of circulating and resident lymphocytes is often missing. For instance, both CD4 and CD8 knockout mice do not show any major deficit in wound healing, despite having reduced cytokine expression and poor macrophage and neutrophil infiltration [125]. Even more surprisingly, athymic nude mice lacking all T cells present scarless skin repair, similar to what happens during fetal development, suggesting that T cells impair re-epithelialization and favor repair by scarring [44]. Opposite data have been obtained in SCID mice transplanted with either CD8^+^, CD4^+^ lymphocytes, or their combination. In this study, CD4^+^ cells, alone or combined with CD8^+^ cells, induced robust re-epithelialization and neoangiogenesis together with reduced inflammation and scarring [45]. While the role of CD8^+^ and CD4^+^ lymphocytes in wound healing in the presence of either tissue ischemia or diabetes has not been investigated in animals, few data are available in humans. Diabetic individuals with foot ulceration showed a decreased number of total circulating lymphocytes [129], with a relative accumulation of activated T cells and increased TNFα levels, indicative of a proinflammatory condition [130]. Overall, this evidence suggests that altered balance of T cell sub-populations may impact on the closure of diabetic wounds, yet further studies are needed to characterize their precise role.

An additional class of αβ-TCR lymphocytes is composed by Tregs. These are considered the major players in immune suppression, maintaining tolerance to self-antigens, and are traditionally characterized by the expression of CD4, CD25, and the transcription factor forkhead box P3 (FOXP3). Emerging evidence indicates that they are a heterogenous population, able to promote tissue regeneration through mechanisms independent from their immune-modulatory function [131,132]. They physiologically reside in both the human and murine dermis [133,134], and rapidly accumulate to the site of wounding. Different receptors have been described as key mediators of their recruitment, including EGFR and CCR4 [135,136]. Depletion of Tregs in healthy mice resulted in delayed wound closure, associated with increased proinflammatory cytokines and macrophage recruitment [137]. Contrasting results were obtained in diabetic mice, in which either Treg depletion or inhibition of their recruitment resulted in increased collagen deposition, reduced cytokine content, and faster wound healing [136]. This again warrants additional studies to better elucidate the mechanisms through which Tregs might modulate the two phases of wounds, and in particular their capacity to control macrophage polarization, as described in the setting of myocardial infarction [138], and revascularization of ischemic tissues [127].

Finally, B lymphocytes have been shown to colonize skin wounds in rodents and humans, but very poor information is available about their function [108,139]. The topical application of naïve B cells, but not T cells, accelerated the closure of either acute or chronic skin lesions in diabetic mice, associated with enhanced collagen deposition, angiogenesis, increased TGF-β, and reduced MMP2 levels [46]. In accordance, in CD19^−/−^mice, B cells are hyporesponsive, and displayed impaired wound healing, decreased recruitment of neutrophils and macrophages, and an altered cytokine profile. On the other hand, transgenic overexpression of CD19, which drives B lymphocyte hyper-responsiveness, induced faster healing [140], thus supporting a positive role of these cells in wound healing. Overall, these findings suggest that a correct balance between the different lymphocyte populations during wound healing may represent a promising mechanism to investigate for future therapies.

## 7. Peripheral Blood Mononuclear Cells

PBMNCs represent a heterogeneous cellular pool composed by roughly 65% lymphocytes, 25% monocytes, and 10% granulocytes [141,142]. They can be easily isolated from venous blood using different methods, including gravity sedimentation, gel layering, filtering and leukapheresis [141,142,143]. 

A few studies have evaluated the potential of PBMNCs, without any cell-specific enrichment, to promote healing when directly applied to the wound. A seminal study in 1994 demonstrated that the topical application of autologous PBMCs to skin ulcers in patients affected by peripheral arterial disease (PAD) and post-thrombotic syndrome resulted in accelerated healing and pain relief [144].

While these cells have not been vastly used to treat chronic wounds in the following years, they were exploited in multiple trials for the treatment of people affected by ischemic diseases, and in particular myocardial infarction (MI). This improved the cardiac function, but their real regenerative potential is still unproven. Starting from the evidence that very few transplanted cells survived in the ischemic heart, an intriguing hypothesis was formulated and assumed that a large proportion of apoptotic transplanted cells could modulate local tissue reaction, by downregulating innate and adaptive immunity [145]. This led to a series of experimental studies supporting the efficacy of the secretome of apoptotic PBMNCs (APOSEC) in improving the outcome of myocardial infarction in rodents [146,147], followed by the application of a similar strategy also in wound healing [148]. A small randomized double-blinded placebo-controlled phase 1 trial on 10 volunteers was performed to establish the safety and the efficacy of autologous APOSEC on artificially created wounds with negative results [49].

A more recent application entails the seeding of a mixture of fibroblasts and PBMNCs in cellular sheets, which can be topically applied to the wound. Incorporation of PBMNCs was shown to produce a variety of cytokines (i.e., VEGF, Hepatocyte Growth Factor, HGF, TGF-β, CXCL1, and CXCL2) and to promote revascularization and wound closure in diabetic mice [47,48].

## 8. Endothelial Progenitor Cells

Highly controversial is the assumption that PBMNC also contains a population of CD34^+^ endothelial progenitor cells (EPCs), which could contribute to vessel formation through their direct incorporation into nascent vascular tubes. This concept was put forward by Asahara in 1997 and assumes that a vasculogenesis-like process might occur in adulthood [149]. The idea was embraced with enthusiasm by the scientific community, as it could pave the way to novel clinical strategies for tissue revascularization.

After more than 20 years of preclinical and clinical trials using EPCs, their real capacity to differentiate into functional endothelial cells and become incorporated into new vessels, providing clinical benefit, has never been proved. Major concerns have been raised on the method used to prove and quantify their differentiation into endothelial cells [150,151,152].

In an attempt to better characterize these cells, CD34^+^ EPCs have been expanded ex vivo and distinguished as early and late EPCs depending on time of appearance of endothelial cells in the culture [150,151,152]. Early and late EPCs appeared to exhibit a distinct phenotype and, thus, they were reclassified as either hematopoietic (CD45^+^/CD133^hi^) or non-hematopoietic (CD45^−^/CD133^lo^) EPCs. Early hematopoietic EPCs do not generate endothelial cells able to form colonies and, when transplanted into lethally irradiated recipient mice, give rise to a minimal number of endothelial cells (no more than 1%) that persist for a few months after transplantation [153,154]. Further analyses revealed that these cells are monocytic in nature and might be assimilated to M2 macrophages, able to secrete proangiogenic molecules. Thus, the only way these cells appear to contribute to angiogenesis is through a paracrine mechanism and not through their direct incorporation into vascular structures. 

In contrast, non-hematopoietic EPCs, forming late endothelial colonies, can be derived from umbilical and peripheral blood but also from other tissues, including white adipose tissue, intestine, and liver [155,156,157,158]. These cells have been variably named over time, until a recent consensus statement on the nomenclature of endothelial progenitors has recommended the use of the term “endothelial colony forming cells” or “ECFCs” [152]. In the following sections, we will still keep the term *EPC*, as authors did not always discriminate between hematopoietic and non-hematopoietic origin in most old studies. The majority of the clinical trials using either hematopoietic *EPCs* or ECFCs have focused on myocardial infarction and peripheral arterial disease, with only a few studies considering the effect of these cells on chronic wounds [55,159]. 

As for other rare populations of circulating cells, the simplest therapeutic strategies consist in either their mobilization from the periphery or their enhanced recruitment at the level of the wound [155]. Pre-clinical studies have largely explored this second strategy. Among the most relevant chemoattractants for *EPCs* is SDF-1/CXCL12, which binds the CXCR4 receptor, widely and constitutively expressed by cells of both hematopoietic and endothelial lineage. This pathway is compromised in diabetic patients, as hyperglycemia is known to reduce SDF1 expression through inactivation of its transcriptional regulator hypoxia-inducible factor-1 alpha (HIF-1α) [50]. In an attempt to rescue the homing of *EPCs* through this pathway, the administration of recombinant SDF-1 into the skin of diabetic mice restored *EPC* recruitment and accelerated wound closure. An alternative strategy consisted in the pharmacological inhibition of dipeptidyl peptidase-4 (DPP4), a membrane-bound extracellular peptidase that cleaves and inactivates SDF-1 [51], with analogous promising results. The clinical translation of these approaches is still at its infancy, with only few human studies showing the actual increase in the number of circulating *EPCs* upon delivery of the DPP4 inhibitor or human recombinant G-CSF (NCT02694575 and NCT01102699). 

Other studies have tried to enhance the *EPC* viability and proliferation at the site of the wound. Starting from the evidence that angiogenesis during the menstrual cycle largely depends on estrogen and that the latter increases the colony-forming capacity of *EPCs* in culture [160,161], the topical administration of estrogen has been successfully validated as a treatment to accelerate wound healing in diabetic mice [52]. However, to what extent this therapeutic effect can be ascribed to *EPCs* or other estrogen-responsive cells participating in the healing process (i.e., keratinocytes and fibroblasts) remains an open question [162,163,164].

Finally, *EPCs* could be directly transplanted into the wound. This has been attempted in multiple preclinical models, using either syngeneic or human *EPCs* in immunocompromised animals [53,54,165]. One trial assessed the effect of the intra-arterial delivery of CD133^+^ EPCs for the treatment of diabetic foot in 53 patients [55]. This treatment prevented any amputation and resulted in a significant increase in limb perfusion, paralleled by increased circulating levels of VEGF-A, and reduced levels of IL-6. Yet, the real clinical benefit derived from this treatment is still not obvious, based on the large standard deviation that is often reported in this type of analysis. In an additional trial, EPCs from diabetic patients with a non-healing foot were first mobilized with G-CSF and then purified as CD34^+^/VEGFR2^+^ cells, prior to their intramuscular injection into the same individuals [56]. Major limitations of the study, i.e., the inclusion of only five patients and the lack of a control group, do not allow any definitive conclusion about the efficacy of this approach.

In conclusion, the persistence of hemangioblasts in postnatal life, able to sustain vasculogenesis-like phenomena in adult organisms, has been harshly challenged. Similarly, *EPCs* of hematopoietic origin have never been reported as being able to give rise to new vessels. It seems more realistic the existence of ECFCs of non-hematopoietic lineage, although the physiological contribution of these cells to the formation of new blood vessels in adult organisms and, more importantly, their real therapeutic potential, still has to be determined. To this end, major efforts are required to define a consensus in terms of their culture methods, phenotypic characterization, therapeutic dose, and route of administration.

## 9. Stromal Vascular Fraction

The evidence that multiple cell types contribute to tissue regeneration in wound healing has prompted the use of heterogeneous cell populations, such as the one that can be obtained from white adipose tissue and is commonly named stromal vascular fraction (SVF). This fraction contains endothelial cells, pericytes, smooth muscle cells, immune cells, fibroblasts, preadipocytes, and a population of multipotent progenitors, often referred as adipose-derived stem cells (ASCs) [166,167]. Being of mesenchymal origin, ASCs differentiate into adipocytes, osteocytes, and chondrocytes and secrete a variety of factors able to sustain tissue regeneration and the formation of new blood vessels in a paracrine manner [168,169,170]. ASCs have been extensively tested for their capacity to accelerate wound healing in multiple models of diabetes, particularly when embedded in scaffolds [171,172]. Notably, the implantation of decellularized scaffolds recapitulates the therapeutic effect, suggesting that the latter relies on the paracrine secretion of pro-regenerative factors. Further studies have shown that ASCs, similar to the whole SVF, exerted a comparable therapeutic effect on wound healing in hyperglycemic mice, resulting in the expression of genes favoring fibroblast migration, angiogenesis, and macrophage recruitment [173], again supporting a paracrine mechanism of action. Similar conclusions were drawn upon the local or systemic delivery of human ASC exosomes, which improved wound healing by promoting fibroblasts’ migration and collagen production [174]. 

Consistent with the immunomodulatory function of other mesenchymal stromal cells, both ASCs and the entire SVF could contribute to wound healing by locally controlling the immune response [175]. For instance, the addition of the SVF to vascularized human dermo-epidermal skin substitutes promoted M2 macrophage polarization at the level of the wound in nude rats [57].

More recently, the idea of using the entire SVF, also including other cell types that can directly contribute to skin regeneration through their direct incorporation into the newly formed tissue, is emerging as a more potent strategy compared to isolated ASCs.

A direct comparison of human SVF with either ASCs or SVF-derived condition medium indicated that the whole SVF was the most effective in promoting angiogenesis, as well as the proliferation and migration of keratinocytes and fibroblasts [176,177]. The superior efficacy of the SVF compared to cultured ASCs was also confirmed by additional studies in nude mice [178].

This indicates that SVF might contribute to tissue regeneration through cellular mechanisms that do not rely exclusively on ASCs or secreted factors. Indeed, fluorescently labeled SVF cells implanted in mouse flanks and ischemic hind limbs were incorporated into newly formed vessels and exhibited an endothelial phenotype, with null contribution from other cell types [179]. 

On the basis of these promising preclinical results, the therapeutic efficacy of the SVF in improving skin regeneration has been investigated in various clinical trials. While most studies have considered a low number of patients, not allowing conclusive results [58,59,60], promising data were obtained by a trial that enrolled 28 patients with diabetic foot ulcers, who were treated with lipoaspirate SVF and compared to a control group of 26 matched patients. The SVF-treated group showed faster healing, resulting in full wound closure at 8 weeks in 100% of patients compared to 62% in the control group. 

Despite these promising results, major concerns limiting the clinical use of the SVF refer to the poor standardization of purification protocols, culture conditions, time, and phenotypic characterization of ASCs and SVF cells. Recent single-cell sequencing approaches are expected to improve the characterization of this heterogeneous cell population and thus the choice of appropriate markers for their purification and therapeutic use [180,181,182,183,184]. 

## 10. Conclusions and Perspectives

The lack of effective therapies to treat non-healing wounds and prevent amputation, together with an increasing understanding of the role of various immune cells in skin regeneration and healing, have promoted the development of cellular and molecular therapies. These essentially aim at mobilizing therapeutic cells into the circulation, recruiting them and modulating their activity at the wound site, or directly supplementing them to the wound bed. However, none of these formulations can be considered a standard of care, likely due to multiple limitations. First, both preclinical and clinical studies often use different parameters to evaluate efficacy. For example, re-epithelialization and revascularization were assessed by multiple modalities and the presence of granulation tissue was variably considered as either a positive or a negative element. Second, clinical end points were mostly based on wound area and healing time, and did not consider either the type of healing (i.e., scarring versus re-epithelialization) or incidence of long-term relapse. Finally, well-designed, randomized, and placebo-controlled clinical trials are still lacking for most cell types.

By assessing the rationale and the supposed mechanism of action of these approaches, it emerges that some signaling pathways regulate the activity of multiple cell types. For example, the SDF1/CXCR4 axis has been exploited to recruit both macrophages and ECFCs, paving the way to the development of multitarget therapies able to orchestrate the participation of different cells. 

This also highlights that a better understanding of the individual contribution of each cell type, in different phases of the healing process, is required to optimize and select the best cellular therapies. New tools will possibly provide simpler models for investigating the role and the therapeutic potential of immune cells in skin regeneration. 

Zebrafish is an emerging model allowing rapid assessment of the healing of skin wounds, which can be easily and reproducibly generated on its flank [27,185,186]. In addition, both angiogenesis and regeneration can be visualized in live imaging through its transparent skin. To what extent this model will be exploitable to test the therapeutic potential of cellular therapies remains an open question. More suitable to this end is the use of ex vivo models of human skin, including two- and three-dimensional cultures of human cells (keratinocytes, fibroblasts, and endothelial cells), organotypic skin cultures, and debrided skin specimens [62,187]. 

## Figures and Tables

**Table 1:** Preclinical and clinical studies based on cell therapy for non-healing wounds.

Cell Type	Approach	Preclinical Data	Clinical Studies
Neutrophils	Modulation	Topical application of miR-129-2-3p on wounds of diabetic mice [28]	-
		Genetic inactivation of PAD4 in diabetic mice [29]	-
	Recruitment	Topical delivery of C15 on burn wounds in mice [30]	-
Monocytes and Macrophages	Recruitment and Modulation	Local expression of SDF-1 through transformed bacteria in mice with diabetes and peripheral ischemia and ex vivo model of human skins [31]	-
	Recruitment	-	Local injection of hGM-CSF [32,33,34]
		-	Local application of β−glucans and MALP-2 [35,36]
	Direct application	-	Local injection of autologous macrophages in diabetic foot [37,38]
	Modulation	Genetic inactivation of MLL1 and Dnmt1 in mice [39,40]	-
Dendritic cells	Modulation	Systemic administration of FL in burn wounds in mice [41]	-
Lymphocytes (DETC)	Modulation	IL-15 administration in diabetic mice [42]	-
Lymphocytes (DETC)	Direct application	DETC engraftment in diabetic mice [43]	-
Lymphocytes (CD4^+^ CD8^+^)		Cell transplantation in athymic nude mice [44] and SCID mice [45]	-
Lymphocytes (B cells)		Topical application in diabetic mice [46]	-
Peripheral blood mononuclear cells	Direct application	Application of cell sheets composed of fibroblasts and PBMCs in diabetic mice [47,48]	Topical application of APOSEC in healthy volunteers [49]
Endothelial progenitors	Recruitment	Local administration of recombinant SDF1 in diabetic mice [50]	Pharmacological inhibition of DPP4 in diabetic wounds [51]
	Modulation	Topical administration of estrogens in diabetic mice [52]	-
	Direct application	Local transplantation of human EPCs in immunocompromised mice [53] or model of burn wound in pigs [54]	Intra-arterial delivery of CD133^+^ EPCs in diabetic foot patients [55]
	Mobilization and Direct application	-	Systemic administration of GM-CSF followed by isolation of CD34^+^/VEGFR2^+^ cells and intramuscular injection in non-healing foot in diabetic patients [56]
Stromal vascular fraction	Direct application	SVF seeding on human epidermal skin substitutes applied in nude rats [57]	Direct application of autologous SVF on diabetic ulcers [58,59] or post-traumatic lower extremity ulcers [60]
